# The effects of an unfamiliar experimenter on proactive and reactive control in children

**DOI:** 10.1038/s41598-025-89193-9

**Published:** 2025-02-18

**Authors:** Aurélien Frick, Clément Belletier, Wenjia Tan, Ning Meng, Qiang Zhou, Stella Christie, Valérie Camos

**Affiliations:** 1 School of Psychology and Neuroscience, University of St Andrews, St Andrews, UK; 2 Laboratoire de Psychologie Sociale et Cognitive (LAPSCO), CNRS, Université Clermont- Auvergne, Clermont-Ferrand, France; 3 Department of Psychological and Cognitive Sciences, Tsinghua University, Beijing, China; 4 Tsinghua Laboratory of Brain and Intelligence, Tsinghua University, Beijing, China; 5 Department of Psychology, University of Fribourg, Fribourg, Switzerland

**Keywords:** Cognitive control development, Audience effects, Proactive and reactive control, Social psychology, Psychology, Human behaviour

## Abstract

Cognitive control development across childhood is critical for later academic achievement. Despite recent advances in the comprehension of how the context influences cognitive control development, no study has ever addressed whether one of the most frequent contextual features of children’s lives (i.e., the presence of another person) impacts control engagement. Here, 123 Chinese children aged 5 and 9 years-old performed, either in the presence of an experimenter or alone, an AX-CPT, a task assessing reactive and proactive control. We found that children were overall negatively affected by the experimenter presence in terms of latencies but not of accuracy. Further, when analysing the trial types separately, we observed that this effect mainly concerned trials requiring children to engage more proactive control and was greater for younger than older children. These results indicate that direct social factors such as the presence of an unfamiliar experimenter seem to modulate cognitive control performance. Future research should continue to examine these effects in the light of the numerous existing social presence theories in order to unravel what are the cognitive mechanisms affected by social presence in childhood.

**Supplementary Information:**

The online version contains supplementary material available at 10.1038/s41598-025-89193-9.

## Introduction

Cognitive control – the goal-directed regulation of thoughts and actions – is critical in children’s everyday lives. For instance, it is involved at school when children have to wait for their turn to talk when the teacher asks a question to the classroom. The development of this ability across childhood predicts various later important outcomes such as health and income^1,2^. Many efforts have thus been done to develop tools improving cognitive control performance, especially in children with lower cognitive control levels, though yielding mixed results (see^3,4^). Recently, more attention has been devoted to better contextualise cognitive control in children, with an important emphasis on how the social and cultural environment influences how children engage cognitive control in both the lab and daily-life settings^5–7^. Yet very little is known about how factors tied to the experimental context influence children’s cognitive control engagement. As such, the present study aimed to provide one of the first examinations of whether the presence (versus the absence) of an unfamiliar experimenter influences cognitive control in pre-schooled and schooled children.

### Social presence effects

The study of the effects of the presence of other(s) on performance (also termed as social facilitation – inhibition) has been one of the first areas of research in psychology. Interestingly, among the first investigations, several were carried out on children aged from 8 to 14 years-old^8,9^. These two studies revealed that literacy and arithmetic school-works were quantitively and qualitatively better when performed in group (also refers to as social increment and social supervaluent, respectively; see^10^). These positive effects were coined as social facilitation by Allport^10^ to refer to a better performance when performing on a task in group than alone. However, in a series of studies on adults, Allport^10^ noted that social facilitation appears to be sensitive to task demands. Indeed, in a simple task such as a free association task in which participants have to write as many words as possible related to a target word, better quantitative and qualitative results were observed in a group situation than in a solitary situation. However, in a more difficult reasoning task, although working in a group still resulted in better quantitative performance (e.g., more arguments made), working alone yielded better qualitative performance (e.g., more thoughtful and deeper arguments). This suggested that there might not be social facilitation when individuals are performing on more cognitively demanding task.

Subsequent works have largely focused on the adult population and have used different social presence types such as individuals working in group on a similar task (i.e., co-action) or individuals being watched by a social agent (i.e., audience), but mostly in non-cognitive tasks (see^11^). Nevertheless, the research using cognitive tasks has yielded several theories (see^12,13^), with three that are often cited in the current literature. The first are drive theories and they are rooted in an influential proposal made by Zajonc^14^, arguing that the presence of others increases arousal and the frequency of dominant responses. This would explain why this presence facilitates performance on easy tasks where correct responses are dominant while it deteriorates performance on harder tasks where incorrect responses are most common. Based on this first account, others have claimed that this arousal is greatly driven by human-specific socialisation such as the feeling of being evaluated^15^, threated^16^, being monitored under uncertainty^17^, which could be further shaped by personality traits^18^.

After mixed support for the drive theories, it has been hypothesised by attention theories that when placed under the presence of someone else, individuals tend to pay attention to both the task and this other person, leading to a greater interference for more cognitively demanding tasks^19^. For Baron^20^ as an example, the presence of others creates a cognitive overload exceeding the attentional capacity. In consequence, when there are few relevant stimuli (easier tasks) the performance increases, but it decreases when there is a higher number of relevant stimuli to pay attention to (harder tasks; see also^21^).

However, other theories have also proposed different types of mechanisms at play in social presence effects, such as feedback loops^22^ or increased effort^23,24^. In the latter case, which echoes motivational accounts^25^, the prepotent response is assumed to be more likely following an increase in effort, leading to predictions generally similar to the drive theories presented above. More precisely, the presence of others would lead to an increase of effort, which would be specific to the prepotent response, leading to faster answers. However, it is to be noted that, if the correctness of the prepotent response is accessible for the participant and if there is enough time, this increase of effort could also lead to a better accuracy for that prepotent response^26^.

### Audience effects on adults’ cognitive control

Of importance, cognitive control tasks appear useful for understanding the cognitive processes underlying social presence, and more specifically audience, effects. Indeed, cognitive control tasks are typically poorly learned and involve few relevant stimuli, which should result into social inhibition or social facilitation, according to the drive theories and attention theories, respectively^27^. For instance, take a well-established cognitive control task, such as the Stroop task^28^, which allows for the computation of the Stroop effect^29^ corresponding to the conflict between a relatively automatic, unintended cognitive process (word reading) and a relatively controlled, intended cognitive process (color naming). Drive theories predict that an audience should increase the Stroop effect, by increasing response latencies to the incongruent words and/or decreasing response latencies to the congruent or neutral trials. Conversely, attention theories lead to the prediction that attentional restriction due to the presence of an audience should help focusing more exclusively on the letter-color cues somewhat at the expense of word reading, thereby reducing the Stroop effect. Consistent with the latter hypothesis, Huguet et al.^30^ found a reduction of the Stroop effect for adult participants placed in a condition where an audience seemed attentive to them (looking at them about 60% of the time spent on the task) as opposed to what was observed for other participants placed in isolation (see^31,32^).

Critically, on more complex cognitive control tasks heavily depending on attentional processes, such as those tapping on cognitive flexibility or working memory, studies have shown that an unfamiliar audience (i.e., the experimenter) impaired performance on these tasks (e.g^33–35^). More specifically, in their experiments, Belletier and Camos^34^ have shown that when attentional processes were not heavily engaged in a working memory task, thus allowing for the use of rehearsal to maintain items in short term memory, there was no negative audience effect on adults’ performance. Conversely, when the adults performed a secondary task consisting in repeating non-sense syllables, therefore impairing rehearsal and calling on the use of attention to maintain memory traces, the presence of the audience impaired recall performance in the working memory task. The authors interpreted this finding as the evidence that the experimenter presence influences working memory through the implication of attentional and executive processes in short-term maintenance, which is in link with brain imaging data showing that an audience leads to active recruitments of brain areas underlying attentional processes^36,37^.

Nevertheless, this audience effect does not affect every adult individuals in the same way. Indeed, this commonly reported negative impact is more pronounced for those with higher cognitive control capacities, and more specifically working memory capacities, who perform below their actual abilities under the experimenter presence. This is contrary to what happens for those with lower capacities who perform similarly whether or not the experimenter remains in the room^27,33^. This can be explained by the fact that the presence of another individual reduces significantly the activity in the prefrontal cortex, which supports cognitive control^38^. Consequently, according to this executive resource hypothesis, the presence of an unfamiliar experimenter has a stronger interference with the cognitive control task at hand for individuals with higher working memory capacity.

However, the presence of another person does not only influence how much cognitive control is engaged,  it also affects how it is qualitatively exerted. According to Braver^39^ dual-mechanism of control framework, individuals engage cognitive control either reactively (i.e., in the moment needed) or proactively (i.e., in preparation and anticipation of an event). To examine the use of these two modes of cognitive control, the gold-standard measure is the AX-CPT^40^. In this task, participants are instructed to make a target keypress when a probe X immediately follows A (i.e., AX trials), and not on AY, BX, BY trials (i.e., when the cue A is not followed by the probe X but by the probe Y, the probe X following the cue B, and the cue and probe other than AX are presented, respectively). Based on these different trial types, reactive control engagement leads to more errors and/or slower response times (RTs) on BX trials than on AY trials, whereas proactive control is associated with more errors and/or longer RTs on AY trials than on BX trials. Using this task, Belletier et al.^41^ replicated that the presence of an unfamiliar experimenter affects more individuals with higher working memory capacity (working memory was measured separately in an alone situation) than those with lower capacity. Indeed, while individuals with higher working memory capacity showed faster responses in proactive control trials when performed alone, the relationship between working memory capacity and RTs reversed under the experimenter presence, with increasing slower responses as a function of increasing higher working memory capacity. These results brought evidence that the experimenter presence undermines proactive control, indicating that social factors affect qualitatively cognitive control, but differently individuals based on their working memory capacity.

### Children’s cognitive control

These different results based on the adult literature can have strong implications for developmental research. Indeed, in experimental settings, children almost always engage cognitive control under the presence of the experimenter, who intervenes either intermittently between test blocks or continuously while children perform a task, to ensure that they correctly understand the instructions. However, very little is known about the effects of an unfamiliar audience (i.e., the experimenter) on these cognitively demanding tasks in children.

Cognitive control develops slowly and undergoes different developmental transitions across childhood^42,43^. One of them relates to children engaging increasingly and adaptively proactive control over reactive control with age (e.g^43–45^). This is particularly due to improvements in working memory capacity, and goal processing and maintenance^46–48^. Whereas it was thought that children younger than 6 years were unable to engage proactive control^49^, there is now consensus regarding that younger children can engage this more demanding form of control under certain conditions^44,50,51^. Importantly, it seems that proactive control is an essential capacity for adaptive controlled behaviours engagement with proactive control being more involved than other cognitive capacities (e.g., sustained attention) in academic skills^52^, and potentially benefitting more than other cognitive control skills from specific metacognitive training^3^. For these reasons, proactive control is becoming the hottest cognitive control ability studied in developmental research.

Given that proactive control is particularly demanding for children, there are reasons to believe that an unfamiliar audience may affect this form of control during childhood. Indeed, some research has shown that providing both social and monetary rewards might encourage children to engage in greater proactive cue preparation (observed at the neural level) but only monetary reward seems to yield larger proactive mobilisation of attention, which is detectable at the behavioural level^53^. Although these results are in line with previous neural and behavioural research on other cognitive control components (e.g^54–56^), these effects on proactive control have not been found at the behavioural level in a recent similar study than Jin et al.^53^ and when children are placed in competitive and cooperative situations^57,58^. However, despite these indications that the social context likely modulates cognitive control in children, it is an open question whether the simple presence of an audience affects differently or similarly cognitive control engagement across development.

### The present study

In the light of what has been developed in the previous sections, the present study examined whether the presence of an unfamiliar audience (i.e., the experimenter) influences cognitive control, and more particularly reactive and proactive control strategies, in 4–5 and 8–9 years-old children. We decided to target proactive/reactive control in this study as these forms of control are increasingly examined in cognitive control studies and more particularly when investigating the influence of social factors^53,57,58^. Moreover, there is also a previous social presence study in adults that has specifically focused on proactive and reactive control^41^. We targeted these two age groups to ensure age-related differences in the use of reactive and proactive control^57,58^ and this could potentially reveal age-related differences in terms of the experimenter presence effects. Here, children performed a short task tapping on working memory capacity (Digit Span) under the presence of an unfamiliar experimenter to control for working memory capacity between groups. Indeed, it has been shown that differences in working memory capacity lead to difference in performance for attentional tasks requiring goal maintenance like the AX-CPT^40^ and that individuals with higher working memory capacity are more likely to choke under experimenter presence^41^. Therefore, there is a need to control for working memory capacity between groups to rule out the possibility that any effect of the experimenter presence might be confounded by group differences regarding this capacity. Then, they performed a child-friendly version of the AX-CPT either in the presence of that experimenter (experimenter presence condition) or in isolation without the experimenter (alone condition). 

Given that no previous work has yet examined how the experimenter presence could affect children’s cognitive control, we drew the following hypotheses based on existing theories, although they were all exploratory. First, we predicted that, in line with the distraction-conflict hypothesis^20^, the experimenter presence should decrease children’s cognitive control performance similar to what is observed in adults. More specifically, in line with the executive resources hypothesis^27^, it might also be predicted that children could engage less proactive control under the experimenter presence than when alone as this form of control requires more executive resources. This effect should be especially present for older children who use this mode of control more consistently and are more sensitive to the context than younger children.

## Results

### Working memory capacity between experimental groups

We checked whether the groups assigned to the different social presence conditions within each age group differed in terms of working memory capacity. Working memory capacity did not differ between groups assigned to the alone and experimenter presence condition in 5-year- olds (*M*_score_ = 3.97, span = 2, and *M*_score_ = 3.93, span = 2, respectively), *t* = 0.06, *p* = .953, and in 9-year-olds (*M*_score_ = 10.27, span = 5, and *M*_score_ = 9.61, span = 5, respectively), *t* = 0.81, *p* = .422. This allowed us to be confident that the groups within each age group were comparable in terms of working memory capacity and discard any alternative interpretation of the findings in terms of differences in working memory capacity.

### All-trials analyses

**Fig. 1:**
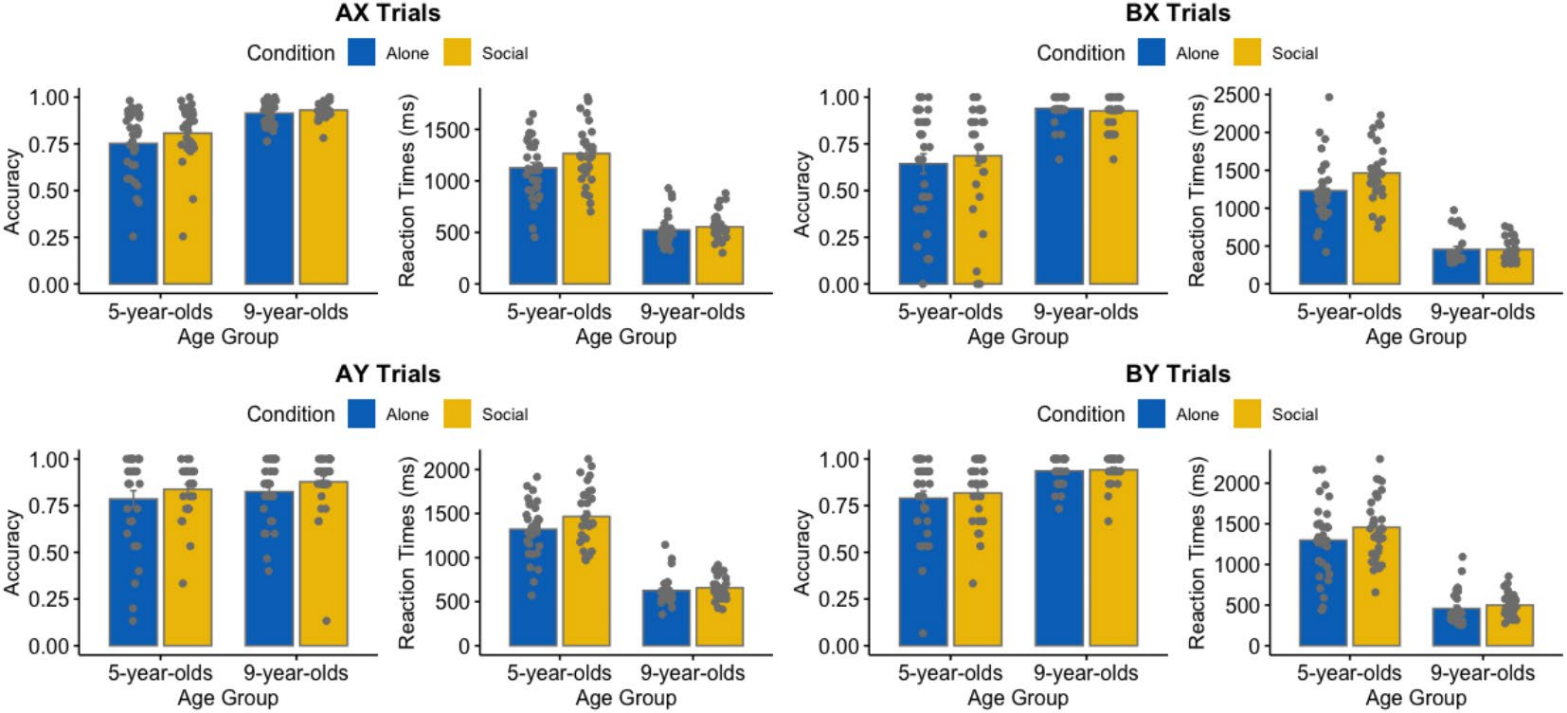
Accuracy and RTs on AX, BX, AY and BY trials as function of age group and social presence condition.

These analyses are represented in Fig. 1.

### Accuracy

For accuracy, the full model controlling for the fact that several experimenters were involved in the data collection (hereafter ‘experimenter control variable’; see the Methods section for more details) was not better than the model without this variable (Model with experimenter control variable: AIC = 2281.2; Model without experimenter control variable: AIC = 2283.0; χ2 = 2.56, *p* = .323). The model without the experimenter control variable yielded main effects of age group, χ2 = 42.56, *p* < .001, and trial type, χ2 = 49.30, *p* < .001, but not of social presence condition, *p* = .268. Age group and trial type significantly interacted, χ2 = 88.65, *p* < .001, and no other interactions were significant *ps* > 0.347. Nine-year-olds were more accurate than 5-year-olds (0.93 vs. 0.80) and children were overall less accurate on BX trials than on AY trials (0.84 vs. 0.87, *p* = .015), which indicated an overall reactive control engagement pattern. However, the age group and trial type interaction revealed that 5-year-olds were less accurate on BX trials than on AY trials (0.69 vs. 0.85, *p* < .001), whereas the opposite pattern was observed in 9-year-olds (0.95 vs. 0.87, *p* < .001). This indicated that for accuracy, younger children engaged more reactive control whereas older children engaged more proactive control (see Table 1 for a depiction of the proactive or reactive control across trial types).

### Response time

For RTs, the full model with the experimenter control variable was not better than the model without this variable (Model with experimenter control variable: AIC = 6509.5; Model without experimenter control variable: AIC = 6509.5; χ2 = 3.95 *p* = .138). The model without the experimenter control variable indicated main effects of age group, χ2 = 152.37, *p* < .001, social presence condition, χ2 = 5.20, *p* = .023, η^2^_*p*_ = 0.04, and trial type, χ2 = 50.80, *p* < .001. Age group and trial type significantly interacted, χ2 = 47.70, *p* < .001, and no other interactions were significant *ps* > 0.107. Nine-year-olds were faster than 5-year-olds (527 ms vs. 1320 ms). Children were slower in the experimenter presence than in the isolation condition (976 ms vs. 871 ms) and were faster in BX than in AY trials (890 ms vs. 1014 ms, *p* < .001), which overall indicated a proactive control engagement pattern (see Table 2). However, the age group and trial type interaction revealed that the difference between BX and AY trials for 5-year-olds was not significant (1336 ms vs. 1388 ms, *p* = .295), while it was significant for 9-year-olds (444 ms vs. 642 ms, *p* < .001).

### Analyses per trial type

The results of these analyses are represented in Fig. 1; Table 1.

### AX trials

For both accuracy and RTs, the full model with the experimenter control variable was not better than the model without this variable (Accuracy: χ2 = 0.324 *p* = .851; RTs: *F*(2, 117) = 1.12 *p* = .331). There was a main effect of age group, χ2 = 48.65, *p* < .001 and χ2 = 136.98, *p* < .001, for accuracy and RTs, respectively. Ten-year-olds (0.92 and 539 ms) were more accurate and faster than 5-year-olds (0.78 vs. 1195 ms). There was also an effect of social presence condition on RTs only, χ2 = 4.28, *p* = .040, η^2^_*p*_ = 0.03, which showed that children were slower under the experimenter presence than when in isolation (910 ms vs. 824 ms), although this effect failed to reach statistical significance for accuracy, *p* = .077. Finally, the interaction between age group and social presence condition was not significant, *ps* > 0.819 and > 0.184 for accuracy and RTs, respectively.

### BX trials

For both accuracy and RTs, the full model with the experimenter control variable was not better than the model without this variable, χ2 = 9.99, *p* = .270, and, F(2,108) = 0.35, *p* = .709.

For accuracy and RTs, there were main effects of age group, χ2 = 59.40, *p* < .001, and χ2 = 124.05, *p* < .001, showing that 9-year-olds were more accurate than 5-year-olds (0.93 vs. 0.62) and faster (451 ms vs. 1345 ms). There were no main effects of social presence condition in both accuracy and RTs, *ps* > 0.052 (effect size for the social presence condition effect on RTs was equal to 0.06). However, there was a significant interaction between social presence condition and age groups for RTs, χ2 = 3.87, *p* = .049, η^2^_*p*_ = 0.03, which revealed that 5-year-olds were slower in the experimenter presence condition than in the isolation condition (1465 ms vs. 1232; *p* = .007) whereas there was no significant difference between these two conditions for 9-year-olds (456 vs. 456; *p* = .999). This interaction was not significant for accuracy, *p* = .489.

### AY trials

For both accuracy and RTs, the full model with the experimenter control variable was not better than the model without this variable, χ2 = 10.57, *p* = .236, and, *F*(1,116) = 2.08, *p* = .129.

Results on accuracy showed that none of the parameters was significant, *ps* > 0.117.

For RTs, there was a main effect of age group, χ2 = 144.98, *p* < .001, which indicated that 9-year-olds were faster than 5-year-olds (641 ms vs. 1394 ms). Social presence condition just failed to reach statistical significance, *p* = .052, η^2^_*p*_ = 0.03. The interaction between age and group and social presence did not reach significance, *ps* > 0.211.

### BY trials

For both accuracy and RTs, the full model with the experimenter control variable was not better than the model without this variable (Accuracy: χ2 = 0.94, *p* = .830, RTs: F(2,114) = 2.134, *p* = .123).

For both accuracy and RTs, age group was significant, χ2 = 31.36, *p* < .001, and, χ2 = 127.28, *p* < .001, indicating that 9-year-olds were more accurate and faster than 5-year-olds (0.94 *vs. .*80 and, 477 ms vs. 1378 ms). Social presence condition and the interaction between age group and social presence condition were not significant in both models, *ps* > 0.091.

**Table 1:** Mean accuracy and mean RTs (and their respective SD) for the two age groups according to the trial type and social presence condition (calculated on full models).

		Experimenter Presence Condition	Alone Condition
5 years-old	AX trials	0.80 (0.02) 1266 (42.2)	0.75 (0.02) 1126 (40.9)
	BX Trials	0.69 (0.02) 1465 (61.2)	0.64 (0.02) 1232 (58.1)
	AY Trials	0.84 (0.03) 1466 (45.9)	0.78 (0.04) 1322 (45.2)
	BY Trials	0.82 (0.02) 1458 (60.6)	0.79 (0.02) 1299 (59.6)
10 years-old	AX trials	0.93 (0.01) 555 (41.6)	0.91 (0.01) 524 (42.2)
	BX Trials	0.93 (0.01) 456 (57.2)	0.94 (0.01) 456 (62.4)
	AY Trials	0.88 (0.03) 658 (45.2)	0.82 (0.03) 626 (45.9)
	BY Trials	0.94 (0.01) 500 (59.6)	0.94 (0.01) 457 (62.7)

## Discussion

The present study examined how social presence, and more especially the presence of an unfamiliar audience (i.e., the experimenter), affects cognitive control in children. To begin with, we replicated well-established age effects on accuracy and RTs for each trial type (except for the accuracy on AY trials), with 5-year-olds being less accurate and slower than 9-year-olds. When the trials were compared to each other, it was found that 5-year-olds showed more a pattern of reactive control engagement, whereas 10-year-olds were observed to engage more proactive control. This pattern of results thus replicates previous findings regarding the developmental transition regarding these modes of control (e.g^57,59^).

But most importantly, we found that the presence and absence of the unfamiliar experimenter did influence children performance. Specifically, when all trials were included in the analyses, both 5-years-old and 10-years-old children were, and across all trials, slower in the experimenter presence condition than in the alone condition. These effects were not observed in the accuracy data as children did not significantly differ between the two social presence conditions. When splitting up the different trial types, we observed that these significant effects on RTs were found only on AX and BX trials, trials where higher accuracy and faster RTs on these trials indicate stronger reliance on proactive than reactive control. But more interestingly, we found that 5-year-olds were slower in the experimenter presence condition than in the alone condition on BX trials, whereas no such difference was observed for 10-year-olds.

These results were partly in line with our hypotheses that predicted to find that children’s cognitive control performance, similarly to adults, would decrease under the experimenter presence than when completing the task alone^41^. Moreover, the significant effects were also confined to RTs, again similar to what is generally observed in adults for conflict and cued tasks (e.g^33,60^), further indicating that social presence effects are greater on response latencies than on response accuracies^12^. This could be potentially due to the fact that this presence interferes with critical attentional resource processes^36,37^ as predicted by attention theories such as the distraction-conflict hypothesis^20^. However, our results appear to be particularly in line with the executive resource hypothesis developed by Belletier et al.^27^, which predicts that the more a given task requires executive resources, the more these resources might be captured by social presence. Indeed, children were slower under the experimenter presence specifically when responding to AX and BX trials. As said previously (see Table 2), faster latencies indicate a stronger reliance on proactive than reactive control. Consequently, slower latencies on these trials under the experimenter presence suggest that this presence might take up important executive resources that are needed to proactively maintain the cue. This interpretation is reinforced by two important findings. First, only 5-year-olds showed slower latencies on BX trials, which are more demanding trials than AX trials, under the experimenter presence. In this condition, these children, who predominantly rely on reactive control, appear to exert a stronger reliance on this less demanding strategy when completing these BX trials. Second, no significant effect of the experimenter presence was observed for AY trials, and no interaction between the effect of this presence and age group was found neither. Given that reliance on reactive control leads to better performance on AY, this seems to further suggest that the presence of the experimenter has no (or little) effect when the task is less demanding in terms of resources.

Nevertheless, one aspect of the executive resource hypothesis predicts that individuals with higher working memory capacity are more likely to choke under the experimenter presence^33,34,41^. One explanation for this would be that these individuals would have enough resources to attend to both the task and the experimenter presence, therefore being more likely to experience interferences from that presence. Based on this rationale, we predicted that older children would be more affected than younger children as the former have more working memory capacities than the latter. However, despite having less working memory capacity, 5-year-olds relied more on a less mature form of control (reactive control) than 10-year-olds on trials requiring important executive engagement (BX trials). Unfortunately, the present study did not allow finer-grained analyses on a potential contribution of working memory capacity as our assessment of this ability was done in the presence of the experimenter whereas it should be ideally done in isolation (see^27^). For now, the data supports the idea that younger children appear to be relying on reactive control to a greater extent in the presence of the experimenter than when alone, especially when the task is challenging to them and requires important attentional and executive resources. Even if this result does not fully fit with the executive resource hypothesis, it is important to note that social presence effects are complex and often modulated by several factors (Garcia-Marques & Fernandes, 2024). For instance, exerting cognitive control on a challenging task could be more unusual for younger than for older children, potentially yielding more evaluative apprehension from the experimenter. However, this is very speculative, and only future research could shed lights on this matter (e.g., asking how children perceive the experimental situation).

The present research provides the first evidence that the simple presence of an unfamiliar experimenter as opposed to their absence in the experimental room modulates cognitive control performance in children, and particularly in younger children when the task is sufficiently challenging and cognitively effortful. Nevertheless, several limitations can be acknowledged that future studies may want to overcome to provide clearer contributions to the field. First, it would be relevant to vary the number of trials (e.g., having a roughly similar number of each trial in the task) and to provide or not (social) feedback after each trial. We believe these two rather simple manipulations could perhaps help precising the contributions of increased arousal and motivation^53,58^. Second, the use of a between-subject design makes the data collection easier as it avoids the participating children to come twice to the lab, which is ideal for social presence studies. Indeed, placing children under social presence in the first place can create carry-over effects (e.g., residuals anxiety or stress) to the next isolation condition, if performed straight after. However, future studies should consider the use of within-subject design to increase power and reduce variability between subjects to capture finer-grained effects. For instance, it is possible that the effects that we found when splitting up the analyses by trial type were not found as such in a larger statistical model including trial type as a factor because of a lack of statistical power to detect relatively small effects (see the observed effect sizes), which were confined to RTs (for similar results in adults, see Belletier et al.^41^. The use of a within-subject design might therefore help to confirm the present findings.

Beyond addressing these limitations, future work should also attempt to deeper our understanding on the effects of the social presence in children’s cognitive control. As Frick (2024) recently argued, the effects of social presence on children’s cognitive control are likely to be influenced by a variety of larger moderated factors, beyond than the social presence itself in the immediate context (e.g., familiarity; Ma et al., 2023). For instance, children who might experience difficulties in school-related tasks (e.g., reading or maths exercises) tapping on cognitive control in their daily lives might be more affected by the presence of an evaluative and unfamiliar figure, particularly if they believe the upcoming task might be too difficult for them. However, to date, little is known regarding to what extent children perceive an unfamiliar experimenter as an evaluative figure and what is the role played by their own past experience and beliefs about their cognitive resources in the effects of others in children’s cognitive control engagement^61,62^.

To conclude, our results suggest that the testing of children by an unfamiliar experimenter in cognitive control research is not without effects. This research contributes to the recent efforts made in this literature to better understand how the environment, could it be physical or social, influences cognitive control development. More particularly, better understanding how the presence of others influences how children engage cognitive control might help precising our theories in the cognitive control but also in the social presence field. For instance, it has been suggested that reporting demographic details might improve theoretical advances from meta-analyses^63^. The present results speak in favour of reporting details about the context of the experimental sessions (e.g., presence of the experimenter, familiarity between the experimenter and the children, place of the testing sessions) as well to contribute to these efforts.

## Methods

### Participants

We recruited 123 children with a roughly equal number (around 30 children) in the two age groups and the two presence conditions: thirty-two 5-year-olds in the alone condition Mage = 4.77, SDage = 0.55, age range = 4.07–5.97, 13 girls) and thirty 5-year- olds in the social presence condition (Mage = 4.64, SDage = 0.43, age range = 4.14–5.76, 16 girls), thirty 9-year-olds in the alone condition (Mage = 8.74, SDage = 0.53, age range = 8.00- 9.91, 15 girls) and thirty-one 9-year-olds (Mage = 8.79, SDage = 0.60, age range = 8.02–9.91, 15 girls) in the social presence condition. We initially ran an a-priori power analysis using G*Power^64^ for General Linear Model analyses (4 groups and 4 measurements, a medium effect size of f^2^ = 0.15 and a desired power of 0.90) that indicated that a minimum sample size of 116.

Children were recruited in Beijing, China. They were all Asian and all, but one, had at least one parent holding a university degree, indicating middle to high socio-economic status. Informed written consent was obtained from caregivers and all children received a small age-appropriate prize (e.g., stickers). This study protocol was approved by the Ethical Committee of Tsinghua University and was in accordance with the guidelines and regulations of Tsinghua University.

### Materials and procedure

Children were individually tested at the laboratory in a single 30-minute session by one of the three trained experimenters. Children were all Mandarin-Chinese native speakers and tested by a native Chinese-speaker experimenter (two females and one male). All children completed a backward digit span task (to control for between-subjects working memory capacity following by a child-friendly version of the AX-Continuous Performance Task (AX-CPT) under one of the two social presence conditions (alone vs. experimenter presence).

### Digit span

To ensure that children within each age group but assigned to the different social presence conditions had a similar working memory capacity, which would rule out any alternative explanations in case of an effect of our social conditions, we administered the digit span task from the WIPPS at the beginning of each session. In this task, children heard a series of digits, pronounced by the experimenter at a pace of one digit per second, that they had to repeat in a backward fashion. Before the test, two demonstrations containing two digits was administered with feedback and guidance from the experimenter, if needed. As instructed in the WIPPS, the test series were administered starting with two series of two digits. If children correctly recalled at least one of the two series, they were administered two new series containing one additional digit. The length of the series increased up to 9 digits or until the children incorrectly recalled both series of any given length. Each time children gave a correct answer, they received one point with a minimum total score of 0 and maximum of 18. A score comprised between 1 and 4 corresponds to a working memory span of 2 (i.e., been able to recall correctly two digits), a score between 5 and 6 to a span of 3 (i.e., three digits) and so on.

### AX-CPT

**Fig. 2:**
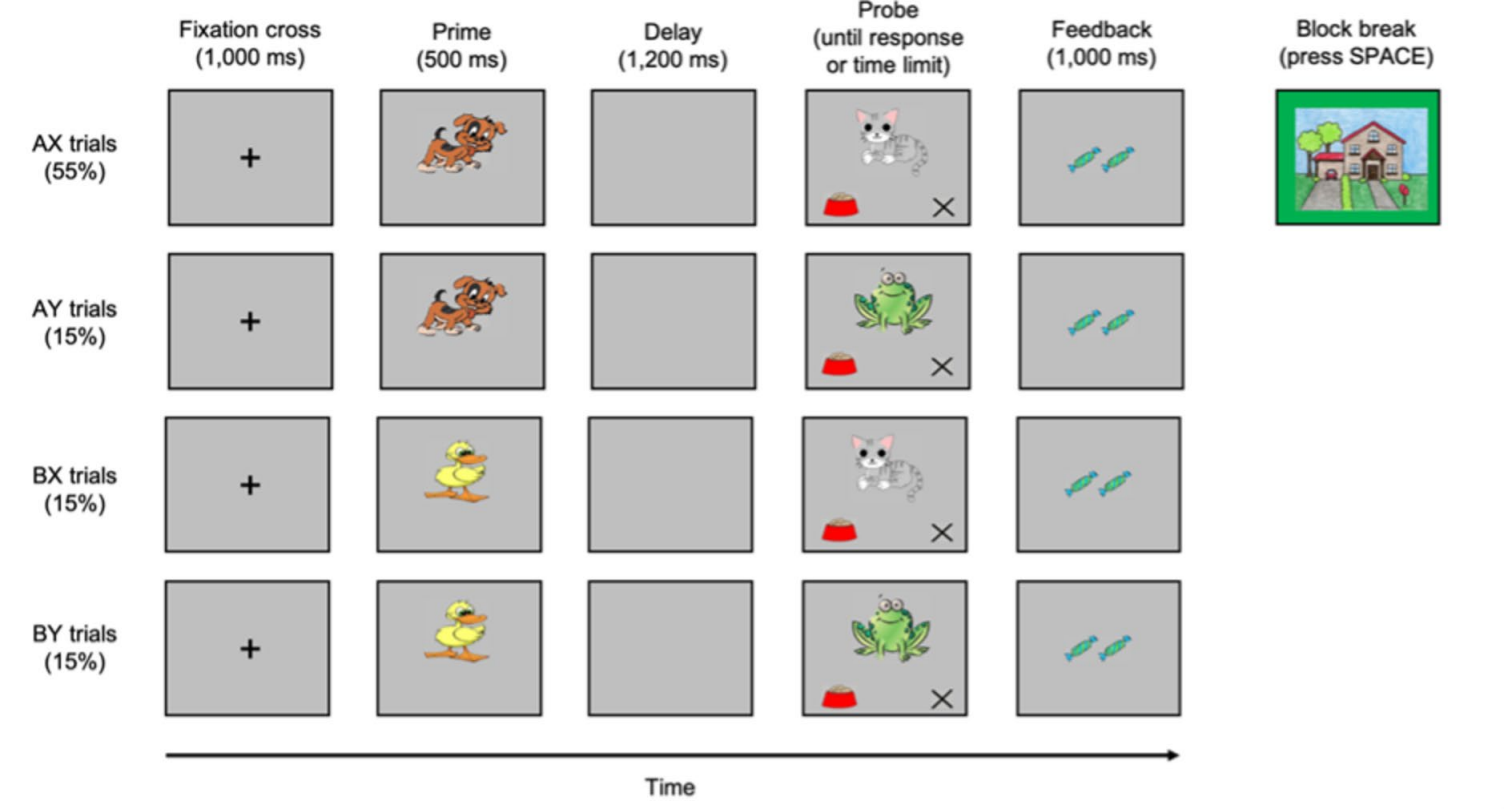
Illustration of each trial type and the block break in AX-CPT used in this study.

All children performed the AX-CPT adapted from Fischer et al. (2018), involving four cartoon animals: a dog, a cat, a frog and a duck. They were instructed to help the dog tell the cat that it is feeding time by pressing the ‘food bowl’ when the dog (A prime) was followed by the cat (X probe; AX trials) and pressing the cross ‘x’ for all other combinations (i.e., dog–frog/AY trials, duck–cat/BX trials, duck–frog/BY trials; (Fig. 2)). The two trials on which proactive control is more efficient are AX and BX trials. For AX trials, individuals have to maintain actively the cue “A” to give a fast response when the target “X” appears (proactive control) rather than giving the response based on the target “X” only (reactive control). For BX trials, individuals have to maintain the incorrect cue “B” in order to give the appropriate response when the target “X” occurs (proactive control). If the incorrect cue is not correctly maintained (hence the use of reactive control), this might lead to more errors. On AY trials, proactive control is less efficient because the active maintenance of the cue “A” leads to wrongly anticipate the correct probe “Y”. On BY trials, the two types of control make no difference between maintaining the incorrect cue “B” (proactive control) or giving a late response based on the incorrect target “Y”. How these trials tap onto proactive and reactive control based on accuracy and response times is summarised in Table 2.

**Table 2:**
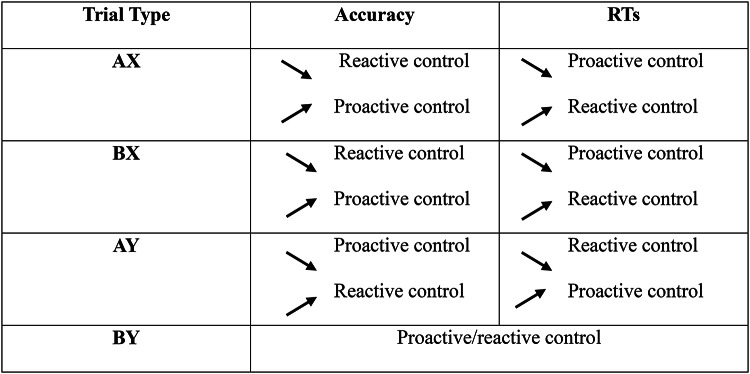
The different engagement of either proactive or reactive control based on increase and decrease of accuracy and RTs across the different trials in the AX-CPT.

Children performed one block of 4 demonstration trials (repeated if one error was made) to make sure they understood the instructions. They then performed 10 practice trials in the presence of the experimenter. These practice trials were used to compute the two time-limits used on all following test trials. The first time-limit (0.75 x mean response time) was used to account for the speed of the response given, above which children received only one candy (to account for slow responses) and below which they received two candies (to account for fast responses). The second time-limit (1.5 x mean response time) was used for a response to be registered in the program. If the response given exceeded this time limit, the response was not registered, and the children received no candy. Following Fischer et al. (2018), we tailored these time limits to each child’s own response time to ensure that the task was equally challenging to all participants given developmental differences between the age groups used in this study in terms of processing and motor speeds. At the end of the practice, children saw a house on a green background and the experimenter indicated to the children that this picture meant that it was a break and that they had to press the space bar to continue the game each time they saw it. Then, children completed 5 blocks, each separated by the house on a green background, of 20 trials under one of the two presence conditions (described below). Each test block contained 11 (55%) AX trials, 3 (15%) AY trials, 3 BX trials, and 3 BY trials (for similar procedures, see Fischer et al., 2018; Troller-Renfree et al., 2020). Children were told that the more virtual candies they will be able to get, the more stickers to keep with them they will receive in exchange.

### Social presence

When performing the AX-CPT, children were randomly assigned to either one of the two experimental conditions. For children assigned to the alone condition, the experimenter left the experimental room after the practice trials. In this condition, the experimenter told the children that they had to work in another room during the time of the task and that they could simply knock on the door to indicate they are finished with the task. Conversely, for children assigned to the experimenter presence condition, the experimenter remained in the room with the child and watched them 60% of the time as traditionally done in adult studies on social presence. For sake of comparability, we implemented the same experimenter presence condition as in other studies^30,41^.

### Data analyses

Data were analysed using R version 4.3.1 (R Core Team, 2023). The data as well as the analytic code are publicly available here: https://osf.io/yvc3k/?view_only=6dc791ea19574c9fbad1f159ffd7ee9d.

Our first analysis was to examine whether the groups assigned to the different social presence conditions within each age group differed in terms of working memory capacity, so the results of the following analyses could not be confounded by differences regarding this capacity. To test this, we ran t-tests comparing the working memory capacity scores of the 5-year-olds assigned to the experimenter presence condition to the 5-year-olds assigned to the alone condition, and similarly for the 9-year-olds.

For the AX-CPT task, we analysed mean accuracy and reaction times (RTs), first across all trials and then within each trial type. The first analyses are in line with the analyses done in most studies while the second set of analyses allows more detailed exploration of the potential effects as there are more AX trials than other trial type in this version of the AX-CPT task and these trials also reflect on different cognitive processes^65,66^. Moreover, this also allowed better comparisons with a previous study that has used the AX-CPT task to examine the influence of the experimenter on cognitive control in adults (Belletier et al., 2019).

Before conducting the analyses, accuracy and RTs data were pre-processed following previous studies. First, responses were considered incorrect if the children responded incorrectly and if they did not provide a response within the time window. Then, for RTs analyses, only correct trials were considered (resulting in the removal of 15.75% of the total trials) and trials below 200 milliseconds (ms) were excluded (0.14% of total correct trials) to account for accidental responses. On these remaining trials, outliers RTs were identified for each participant and each trial type if they were above 2.5 above the median^67^. We computed the means of correct responses and RTs for each trial type.

### All-trials analyses

Accuracy data was proportional ranging from 0 to 1. Therefore, for all trials analyses, data were analysed using General Linear Mixed Models and set to fit a binomial family structure (link=’logit’). For RTs data, we compared the fit of different distributions (namely the Gaussian and Gamma distributions after visual inspections) using the *fitdistriplus* package^68^, which revealed that the Gaussian distribution provided the better fit for all types of trials. GLMM and LMM were fitted with age group (5-year-olds, 9-year-olds) and social presence condition (experimenter presence, alone) as between-subject factors, and trial type (AX, BX, AY, BY) as within-subject factor.

Our analyses strategy started by fitting two null and two full models comprising either only the random effect of Subject, or all main effects, their interactions and the random effect item, respectively. One of the null and and one of the full models contained an experimenter control variable, to account for the unbalance number of children that each experimenter tested, and the two remaining models did not include this experimenter control variable. The comparison between these models led to exclude the experimenter control variable variable in the full and reduced models if both the null and full models containing this control variable did not fit significantly better the data than the respective models without this variable. Once the full model was fitted, we verified using the *performance* package that there was no over-dispersion resulting from this full model for the two GLMM and LMM. We fitted two reduced models for both accuracy and RTs that comprised only the main predictors and all the 2-way interactions. We checked collinearity on the model that contained the main effects only using the *performance* package again, and no collinearity was observed. To test the significance of the parameters, we used the drop1 function, and compared the full model with the corresponding reduced models that lacked the predictor of interest to avoid increased type 1 error risk resulting from multiple testing. Pairwise comparisons were used with Tukey adjustments when there were multiplicity issues using the *emmeans* package^69^. Finally, we computed partial eta-squared using the package *effectsize*^70^. Plots were generated with the package *ggplot2*^71^.

### Each trial analyses

Following these all-trials analyses, we ran analyses on each trial separately. Our analytic approach was similar, except that we fitted General Linear Models (GLMs) to account for the between-subject structure of these analyses. For the accuracy data, we initially fitted these models using a binomial (link = ‘logit’) family structure. However, our assumption checks revealed significant overdispersion in the data resulting from these models. We therefore fitted these models using a quasibinomial (link = ‘logit’) structure to adjust for these overdispersions. For RTs, as the Gaussian distribution was always most appropriate, these GLMs were fitted using this family structure. We also checked whether adding the experimenter control variable would improve or not the different models by comparing the null and full models with this control variable to a corresponding model without that variable.

## Electronic supplementary material

Below is the link to the electronic supplementary material.

Supplementary Material 1

## Data Availability

The data as well as the pre-processing and analytic code are available in the Open ScienceFramework repository at https://osf.io/yvc3k/?view_only=6dc791ea19574c9fbad1f159ffd7ee9d.
