# Supplementary material for: The effects of an unfamiliar experimenter on proactive and reactive control in children
Source: Sci Rep. 2025 Feb 18;15:5860. doi: 10.1038/s41598-025-89193-9 (PMC11836422; doi:10.1038/s41598-025-89193-9)
Supplement: Supplementary file 1 — Supplementary Material 1 [file 41598_2025_89193_MOESM1_ESM.docx]

**Supplemental Material**

For these analyses, we combined accuracy and RT data using the Inverse Efficiency Score (IES) developed by Townsend and Ashby, 1983). This measure is obtained by dividing the mean RT of correct responses by the proportion of correct responses for each individual. We analysed IES separately for each trial type using the similar analytic procedure than in the main manuscript (see Methods section).

*AX trials*

There were main effects of there were main effects of age group, χ2 = 97.68, p < .001, but not of social presence condition, p = .737, and not interaction between these terms, p = .920. 5 year-olds had a higher IES than 10-year-olds (1647 ms vs. 585 ms).

*BX trials*

There were main effects of there were main effects of age group, χ2 = 29.09, p < .001, but not of social presence condition, p = .508, and not interaction between these terms, p = .920. 5 year-olds had a higher IES than 10-year-olds (2349 ms vs. 503 ms).

*AY trials*

There were main effects of there were main effects of age group, χ2 = 52.82, p < .001, but not of social presence condition, p = .991, and not interaction between these terms, p = .752. 5 year-olds had a higher IES than 10-year-olds (1871 ms vs. 799 ms).

*BY trials*

There were main effects of there were main effects of age group, χ2 = 84.34, p < .001, but not of social presence condition, p = .380, and not interaction between these terms, p = .576. 5 year-olds had a higher IES than 10-year-olds (1824 ms vs. 516 ms).

**References**

Townsend, J. T. (1983). Stochastic modeling of elementary psychological processes. *CUP Archive*
